# Cholecystokinin type B receptor-mediated inhibition of A-type K^+^ channels enhances sensory neuronal excitability through the phosphatidylinositol 3-kinase and c-Src-dependent JNK pathway

**DOI:** 10.1186/s12964-019-0385-8

**Published:** 2019-06-18

**Authors:** Shumin Yu, Yuan Zhang, Xianyang Zhao, Zhigang Chang, Yuan Wei, Yufang Sun, Dongsheng Jiang, Xinghong Jiang, Jin Tao

**Affiliations:** 1 Department of Physiology and Neurobiology & Centre for Ion Channelopathy, Medical College of Soochow University, 199 Ren-Ai Road, Suzhou, 215123 People’s Republic of China; 2 Department of Geriatrics and Institute of Neuroscience, the Second Affiliated Hospital of Soochow University, Suzhou, 215004 People’s Republic of China; 3 Department of Intensive Care Unit, Beijing Hospital Ministry of Health, Beijing, 100730 People’s Republic of China; 4 Comprehensive Pneumology Center, Helmholtz Zentrum München, 81377 Munich, Germany; 5 Jiangsu Key Laboratory of Neuropsychiatric Diseases, Soochow University, Suzhou, 215123 People’s Republic of China

**Keywords:** Cholecystokinin, A-type K^+^ channel, Dorsal root ganglion, Neuronal excitability

## Abstract

**Background:**

Cholecystokinin (CCK) is implicated in the regulation of nociceptive sensitivity of primary afferent neurons. Nevertheless, the underlying cellular and molecular mechanisms remain unknown.

**Methods:**

Using patch clamp recording, western blot analysis, immunofluorescent labelling, enzyme-linked immunosorbent assays, adenovirus-mediated shRNA knockdown and animal behaviour tests, we studied the effects of CCK-8 on the sensory neuronal excitability and peripheral pain sensitivity mediated by A-type K^+^ channels.

**Results:**

CCK-8 reversibly and concentration-dependently decreased A-type K^+^ channel (*I*_A_) in small-sized dorsal root ganglion (DRG) neurons through the activation of CCK type B receptor (CCK-BR), while the sustained delayed rectifier K^+^ current was unaffected. The intracellular subunit of CCK-BR coimmunoprecipitated with Gα_o_. Blocking G-protein signaling with pertussis toxin or by the intracellular application of anti-G_β_ antibody reversed the inhibitory effects of CCK-8. Antagonism of phosphatidylinositol 3-kinase (PI3K) but not of its common downstream target Akts abolished the CCK-BR-mediated *I*_A_ response. CCK-8 application significantly activated JNK mitogen-activated protein kinase. Antagonism of either JNK or c-Src prevented the CCK-BR-mediated *I*_A_ decrease, whereas c-Src inhibition attenuated the CCK-8-induced *p*-JNK activation. Application of CCK-8 enhanced the action potential firing rate of DRG neurons and elicited mechanical and thermal pain hypersensitivity in mice. These effects were mediated by CCK-BR and were occluded by *I*_A_ blockade.

**Conclusion:**

Our findings indicate that CCK-8 attenuated *I*_A_ through CCK-BR that is coupled to the G_βγ_-dependent PI3K and c-Src-mediated JNK pathways, thereby enhancing the sensory neuronal excitability in DRG neurons and peripheral pain sensitivity in mice.

## Background

Cholecystokinin (CCK), a gastrointestinal polypeptide hormone existing in a variety of amino acid chain lengths, has been isolated from the central nervous system and peripheral tissues [1]. Two types of functional membrane receptors, cholecystokinin A receptor (CCK-AR), located mainly on pancreatic acinar cells, and CCK-BR, mostly in the stomach and nervous system tissues, have been identified as the endogenous receptors of CCK [2]. CCK, acting through its receptors, is found to be involved in the regulation of a variety of physiological functions in the nervous system, including metabolic, neurotrophic and modulatory actions [3]. Additionally, in vitro experiments have suggested that CCK might regulate the sensitivity of nociceptive sensory neurons [4], where CCK-BRs were abundantly expressed [5, 6]. It has been established that the elevated level of CCK mRNA in the dorsal root ganglia (DRG) induced by peripheral nerve injury sensitizes and excites primary afferent sensory neurons, leading to pain hypersensitivity [7], with the application of CCK inducing pronociceptive effects [8]. Evidence also suggests that CCK is a potential trigger for increased visceral sensitivity in healthy subjects [9] as well as in irritable bowel syndrome patients [10, 11]. Moreover, antagonism of CCK receptors can effectively reverse burn-induced mechanical allodynia [5], and the deletion of the CCK-BR gene attenuates the symptoms of mechanical allodynia in neuropathic pain [12]. So far, however, the mechanisms underlying the CCK-mediated hyperalgesia still remain unclear.

Changes in neuronal excitability of peripheral sensory neurons might directly regulate symptoms of pain, such as allodynia, hyperalgesia and spontaneous pain [13]. Voltage-gated K^+^ channels (Kv) are one of the major classes of ion channels responsible for driving neuronal excitability in both the central and peripheral nervous system [14] and in whole-cell patch clamp recordings are separated into two major categories: a large transient component characteristic for fast-inactivating A-type channels and a sustained delayed-rectifying channels, which respectively mediate *I*_A_ and *I*_DR_ currents [15, 16]. A-type channels are sensitive to millimolar concentrations of 4-aminopyridine (4-AP) [17], and they play pivotal roles in the control of the electrical properties and excitability of nociceptive neurons [14, 15]. Recent evidence has suggested that A-type channels have been implicated in pain plasticity and neuropathic conditions [17], which begin with the aberrant firing of action potential bursts in damaged neuronal tissue. For example, peripheral nerve injury results in the reduction of channel expression, thereby decreasing *I*_A_, and enhancing the sensory neuronal excitability and pain sensitivity [18, 19]. Manipulation of *I*_A_ is, therefore, predicted to affect neuronal excitability and useful for pain treatment.

In the current study, we examined the regulation of CCK-8 on *I*_A_ in small-sized (< 30 μm in soma diameter) DRG neurons to determine whether A-type K^+^ channels mediate the nociceptive actions of CCK-BR.

## Materials and methods

### Animals

Adult ICR mice (male, 6–8 weeks of age) were purchased from the Experimental Animal Center of Soochow University. Mice were maintained in specific-pathogen-free facilities on a 12-h light-dark cycle at a room temperature of 22 ± 1 °C, and housed in cages with access to food and water ad libitum. All animal studies were conducted in accordance with the National Institutes of Health’s Guidelines for Animal Care and Use and approved by the Animal Care and Use Committee of Soochow University.

### Isolation of DRG neurons

DRG neurons were obtained from lumber L4–6 segments in adult ICR mice (male, 6–8 weeks of age) using enzymatic dissociation procedure as described previously [20, 21]. Dissociated DRG neurons were plated onto Matrigel-coated glass coverslips and maintained in incubators until recording. We sorted DRG neurons into groups based on the soma diameter distribution of small (< 30 μm in soma diameter), medium (30–40 μm in soma diameter), and large-sized (> 40 μm in soma diameter) DRG neurons [22], and in patch-clamp experiments we only recorded the small-sized neurons. Cells were subjected to whole-cell recordings within 24–48 h of plating.

### Electrophysiological recordings

Whole-cell patch-clamp recordings were performed at room temperature (23 ± 1 °C). Recording electrodes (World Precision Instruments, USA) pulled from borosilicate glass microcapillary tubes (Sutter Instruments) had resistances from 3 to 5 MΩ when filled with internal solution. Recordings were made using a MultiClamp 700B patch-clamp amplifier (Molecular Devices, USA). Whole-cell currents were low-pass filtered at 2–5 kHz. The values of cell capacitance and series resistance were taken directly from readings of the amplifier after electronic subtraction of the capacitive transients. In voltage-clamp mode, 80% of the series resistance was electronically compensated. A P/6 protocol was used for on-line leak subtractions. The external solution used for Kv current recordings contained (mM): KCl (5), choline-Cl (150), MgCl_2_ (1), CaCl_2_ (0.03), glucose (10) and HEPES (10) adjusted to pH 7.4 with KOH, 310 mOsm. The internal solution used for Kv current recordings contained (mM): KCl (140), Na-GTP (0.3), Mg-ATP (3), 0.5 CaCl_2_, MgCl_2_ (1), EGTA (5) and HEPES (10) adjusted to pH 7.4 with KOH, 295 mOsm. The external solution used for Cav current recordings contained the following (mM): BaCl_2_ (5), tetraethylammonium chloride (TEA-Cl) (140), CsCl (5), HEPES (10), MgCl_2_ (0.5) and glucose (5.5) adjusted to pH 7.35 with TEA-OH, 310 mOsm. The internal solution used for Cav current recordings contained the following (mM): CsCl (110), Mg-ATP (4), EGTA (10), Na-GTP (0.3) and HEPES (25) adjusted to pH 7.4 with CsOH, 295 mOsm. To separate T-type channel currents, we applied the L-type channel blocker nifedipine (5 μM) and the N- and P/Q-type channel blocker ω-conotoxin MVIIC (0.2 μM) in the external solution. The external solution used for both Nav current and current-clamp recordings contained the following (mM): KCl (2), MgCl_2_ (2), CaCl_2_ (2), NaCl (128), HEPES (25) and glucose (30) adjusted to pH 7.4 with NaOH, 305 mOsm. The internal solution contained the following (in mM): NaCl (10), KCl (110), Na-GTP (0.3), Mg-ATP (4), EGTA (2) and HEPES (25) adjusted to pH 7.3 with KOH, 295 mOsm. Path pipettes had resistance from 2 to 3 MΩ for internal dialysis with compounds. Monoclonal antibodies raised against Gα_o_ (anti-G_o_-Ab, Santa Cruz Biotechnology) or Gα_i_ (anti-G_i_-Ab, Santa Cruz Biotechnology) were diluted to 1: 100 times from a stock solution (200 μg/ml). This antibody used in the present study has been previously shown to specifically recognize the G-protein Gα_o_ but not Gα_i_ subunit [20, 23]. For the whole cell experiments, the antibody was diluted into the intracellular solution and loaded into the pipette. In patch-clamp recording in which cells were dialyzed with inhibitors or activators, protocols were initiated at least 5 min after breaking the membrane patch.

### Immunoblotting

Immunoblot analysis was conducted as described in our previous studies [23]. Briefly, equal amounts of proteins (25 μg) were separated by 10% SDS–PAGE and electroblotted onto PVDF membranes (Merck Millipore, Germany). Blotted proteins were probed with the following primary antibodies: goat anti-CCK-AR (1: 500, Abcam), goat anti-CCK-BR (1: 500, Abcam), rabbit anti-phospho-Akt (1:2000, Cell Signaling Technology), rabbit anti-Akt (1:1000, Cell Signaling Technology), rabbit anti-phospho-p38 MAPK (1:1000, Cell Signaling Technology), rabbit anti-p38 MAPK (1:1000, Cell Signaling Technology), rabbit anti-phospho-ERK1/2 (1:2000, Cell Signaling Technology), rabbit anti-ERK1/2 (1:1000, Cell Signaling Technology), rabbit anti-phospho-SAPK/JNK (1:1000, Cell Signaling Technology), rabbit anti-SAPK/JNK (1:1000, Cell Signaling Technology), rabbit anti-phospho-Src (pTyr418, 1:2000, Abcam), rabbit anti-Src (1: 1000, Abcam) and rabbit anti-GAPDH (1:5000, Abcam). After extensive washing in TBST, membranes were incubated with horseradish peroxidase-conjugated anti-rabbit or anti-goat IgG (1: 8000, Multi Sciences). Chemiluminescent signals were generated using a SuperSignal West Pico trial kit (Pierce) and detected using ChemiDoc XRS System (Bio-Rad Laboratories). The software Quantity One (Bio-Rad Laboratories) was used for background subtraction and for quantification of immunoblotting data.

### Co-immunoprecipitation (co-IP)

Total cellular proteins were extracted using homogenization buffer (Tris 20 mM, pH 7.4, NaCl 150 mM, EDTA 1 mM, 0.5% Triton, and DTT 1 mM) supplemented with a cocktail of protease inhibitors. After centrifugation at 21,000 *g* for 30 min at 4 °C, the supernatant was saved and protein concentration was measured by BCA protein assay (Beyotime, Shanghai, China). Extract containing 500 μg of protein was incubated at 4 °C for 3 h with 3 μg of goat polyclonal antibody against CCK-BR (1: 500, Abcam). Protein A-Sepharose beads (Amersham Biosciences) were added to the samples and gently shaken for 4 h at 4 °C. Beads were then rinsed and removed in lysis buffer. The pellet was boiled with 4 × Laemmli sample buffer and separated by SDS-PAGE. Immunoreactive proteins on membranes were developed as described above.

### Immunofluorescence staining

Immunohistochemistry was performed as previously described [20, 23]. Tissue samples were sectioned into 15 μm thin slices using a cryostat (CM 1950; Leica, San Jose, USA). The sections were blocked with 5% normal goat serum in PBS, plus 0.2% Triton X-100 for 1 h at room temperature then incubated overnight at 4 °C with primary antibody against CCK-BR (goat, 1: 500, Abcam), antibody against NF-200 (mouse, 1:1000, Abcam), or antibody against CGRP (mouse, 1:500, Abcam). Sections were washed three times with PBS at room temperature, followed by Cy3-conjugated donkey anti-goat IgG (1:500, BBI Life Science), FITC-conjugated donkey anti-mouse IgG (1:200, BBI Life Science) or FITC-IB4 (1:200, Sigma) in PBS at room temperature for 2 h. After sections were washed three times with PBS at room temperature, images were captured with a fluorescence microscope (Nikon 104c, Japan). Negative controls, omitting each primary antibody, were used in each case, and no significant staining was observed in these samples (data not shown).

### PKA activity assay

PKA activity in homogenates was determined by enzyme-linked immuno sorbent assay (ELISA, Promega), according to the manufacture’s instructions. Briefly, the cells were pretreated with either vehicle or KT-5720 for 30 min, followed by treatment with either vehicle (0.1% DMSO), or forskolin for 15 min. The cells were washed with ice-cold phosphate-buffered saline (PBS), placed on ice, and incubated with 200 μl lysis buffer. After a 10-min incubation on ice, the cells were transferred to microcentrifuge tubes. Cell lysates were centrifuged for 15 min, and aliquots of the supernatants containing 0.2 μg of protein were assayed for PKA activity. The activity is expressed as RLU^− 1^ (relative light units)/amount of protein.

### PI3K activity assay

Cells were stimulated with or without CCK-8 (100 nM) for 15 min. After stimulation, PI3K activity in homogenates was determined with a PI3-Kinase HTRF™ Assay kit (Millipore Corporation, Bedford, MA), using 20 μg of protein for each sample, as stated in the manufacturer’s protocol. HTRF was then measured with an excitation wavelength of 335 nm and emission wave length of 620 and 665 nm with a spectrofluorometer (Tecan, Infinite M1000, Salzburg, Austria).

### Behavioral test

Behavioral testing was conducted in an appropriately lighted, quiet room, always during the light cycle between 9:00 AM and 4:00 PM in a series and by the same experimenter. The operator who performed the behavioral tests was blinded to all treatments. Animals were allowed to acclimate to a testing room for at least 30 min before performing the assessments. Mechanical sensitivity was determined on paw withdrawal to manual application of graded von Frey hairs (0.02–2.56 g; Stoelting) to the plantar surface as described previously. Thermal sensitivity was tested using a commercially available paw thermal stimulation system (IITC Life Sciences), and are expressed as paw-withdrawal latency (PWL) and tail-flick latency. Animals were gently dropped into an acrylic box with a metal floor that was preheated to a certain temperature. The values of PWL were calculated using a timer that was started when the animal is released onto the preheated plate and stopped at the moment of withdrawal, shaking, or licking of either hind paw. The cutoff latency was set to prevent tissue damage. All animals were tested once for each temperature per session in a random sequence. All drugs or vehicle were injected subcutaneously into the plantar surface of one hind paw in a volume of 10 μl. The pH of the solutions was adjusted at 7.4 to prevent skin irritation.

### Materials

All drugs were purchased from Sigma (MO, USA) unless otherwise indicated. Stock solutions of 4-aminopyridine (4-AP), pertussis toxin (PTX), cholera toxin (CTX), PMA (Tocris Bioscience, Ellisville, MO) and ω-conotoxin MVIIC (Tocris Bioscience, Ellisville, MO) were prepared in distilled deionized water. Z941 was a kind gift from Dr. Terrance P. Snutch (University of British Columbia, Vancouver, British Columbia, Canada). Stock solutions of cholecystokinin-8 (Tocris Bioscience, Ellisville, MO), LY294002, CCK-4, nifedipine, forskolin, gallein, wortmannin, KT5720 (RD system), devazepide, LY225910, GW5823, BC264, SP600125, SB203580, anisomycin, PP2, PP3, Akt inhibitor III, U0126, and GF109203X were prepared in dimethylsulfoxide (DMSO). The final concentration of DMSO in the bath solution was estimated to be less than 0.01%, and this compound had no functional effects on *I*_A_ (not shown).

### Data analysis

In electrophysiological experiments, data acquisition and analysis were performed with Clampfit 10.2 (Axon Instruments) and/or GraphPad Prism 5.0 software (Prism Software). The amplitude of *I*_A_ was measured at the peak. The data plots were fit by the Boltzmann equation for the activation and inactivation curves as described previously [24]. All data are presented as means ± SEM. Statistical significance between two groups was determined using a paired or unpaired Student’s *t* two-tailed test. Comparisons of multiple groups against a pooled control were tested using one-way analysis of variance (ANOVA) followed by a Bonferroni’s post-test. Differences in values over time among groups were done using two-way ANOVA. The criterion for significance in all analyses was considered as *p* < 0.05.

## Results

### CCK-8 selectively decreased *I*_A_ in DRG neurons

**Fig. 1:**
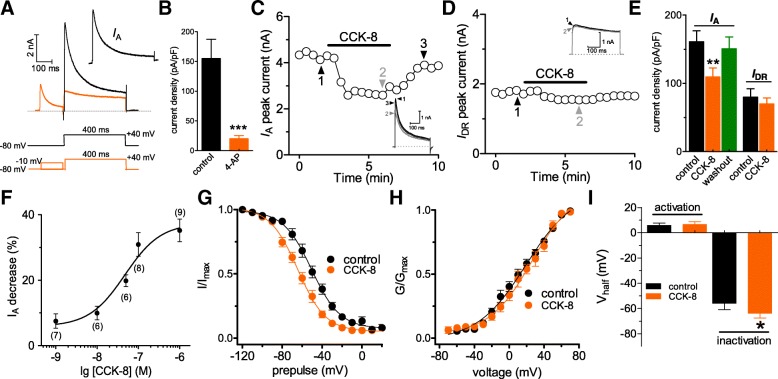
CCK-8 selectively decreased *I*_A_. **a**, isolation of *I*_A_ in mouse DRG neurons. *I*_A_ was isolated by digital subtraction of current traces in which the transient outward K^+^ current had been inactivated by a brief delay at − 10 mV (100 ms, see *Insets*) from corresponding current traces without such a delay. This two-step voltage protocol was used for the isolation of *I*_A_ indicated in all subsequent experiments. **b**, effects of 5 mM 4-AP on the current density of *I*_A_ (*n* = 6). The current density was calculated as the ratio of peak current to membrane capacity (pA/pF). **c, d** time course of changes in *I*_A_ (**c**) or *I*_DR_ (**d**) peak amplitude mediated by 100 nM CCK-8. *Inset*s show the exemplary current traces. The Arabic numerals indicate the relative points utilized for exemplary current traces. **e**, bar graph showing that 100 nM CCK-8 selectively decreased the current density of *I*_A_ indicated in panels *B* (*n* = 8) and *C* (*n* = 10) respectively. **f** a concentration-response curve for CCK-8 is displayed. Mean values on concentration-response curves were fitted to the sigmoidal *Hill* equation: PD ([CCK-8]) = PD_max_/ (1 + (EC_50_/[CCK-8])^n^), where PD_max_ is the maximal percent decrease of peak *I*_A_, EC_50_ is the concentration that produces half-maximum effect occurs and *n* is the Hill coefficient. Cell numbers at each concentration were shown in round brackets. **g, h**, CCK-8 did not significantly alter the steady-state activation curve of *I*_A_ (*n* = 9, **g**), but shifted the steady-state inactivation curve of *I*_A_ leftward (*n* = 12, **f**). **i**, summary data showing the effects of 100 nM CCK-8 on V_*half*_ of the activation and inactivation curves. Voltage-dependent activation was measured with voltage commands ranging from − 70 to + 70 mV (400 ms, in 10 mV increment). Steady-state voltage-dependent inactivation of *I*_A_ was determined by varying a 150-ms conditioning prepulse from − 120 to + 20 mV followed by a 500-ms voltage step pulse to + 40 mV. *p < 0.05, **p < 0.01 and ***p < 0.001 vs. control

The studies in vitro of nociceptive processing usually examined different subtypes of peripheral sensory neurons [25, 26]. In the present study, we limited patch-clamp recordings to small-sized DRG neurons (< 30 μm in soma diameter) as these neurons are primarily involved in nociceptive signaling [19, 26]. Two main types of outward voltage-gated K^+^ channel (Kv) currents have been characterized in these nociceptive neurons — the transient A-type K^+^ channel currents (*I*_A_) and the sustained and delayed-rectifier K^+^ channel currents (*I*_DR_) [15, 16]. We first isolated these two kinetically different whole-cell currents. A total outward current exhibiting a rapidly inactivating and a more sustained component was elicited by a depolarizing pulse from the holding potential of − 80 mV to + 40 mV (Fig. 1a). Biophysical separation of a delayed-rectifier current (*I*_DR_) was obtained by a depolarizing prepulse to − 10 mV, which inactivated the transient channels. *I*_A_ was then isolated by subtracting *I*_DR_ from the total current (Fig. 1a). Addition of 5 mM 4-aminopyridine (4-AP) inhibited the remaining outward current by 87.1 ± 5.3% (*n* = 6, Fig. 1b), further confirming the effective isolation of *I*_A_.

Application of 100 nM CCK-8 to small-sized DRG neurons significantly decreased *I*_A_ by 30.9 ± 3.7% (*n* = 8, Figs. 1c and e), while *I*_DR_ was not effectively affected (decreased by 1.2 ± 0.9%, *n* = 10, Figs. 1d and e). The amplitude of *I*_A_ partially recovered after CCK-8 washout (Fig. 1c). The CCK-8 effect on *I*_A_ was concentration-dependent (Fig. 1f). The half-maximal inhibitory concentration (IC_50_) calculated from a sigmoidal *Hill* equation [23, 24] observed at 47.3 nM (Fig. 1f). Further, we examined whether CCK-8 would alter the biophysical properties of *I*_A_. While no significant changes were observed in the activation properties of *I*_A_ (V_*half*_ from 5.8 ± 1.6 mV to 6.5 ± 2.5 mV, *n* = 9, Figs. 1h and i), CCK-8 shifted the steady-state inactivation curve to the hyperpolarized level by 7.8 mV (V_*half*_ from − 55.9 ± 3.9 mV to − 63.7 ± 2.8 mV, *n* = 12, Figs. 1g and i). These findings reveal that the CCK-8-induced reduction in *I*_A_ is mainly contributed by an increased proportion of channels retained in the inactivated state.

### The CCK-BR mediated the CCK-8–induced *I*_A_ decrease

**Fig. 2:**
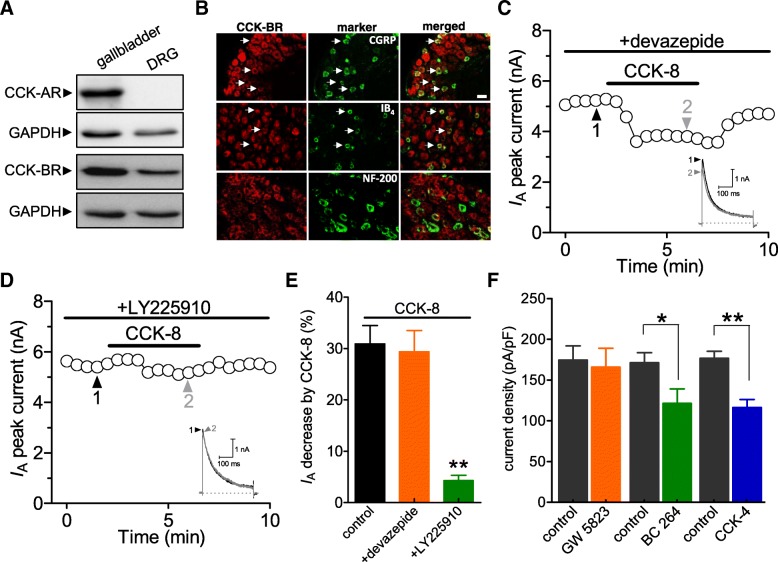
CCK-8 decreased *I*_A_ via activation of CCK-BR. **a,** western blot analysis demonstrated the expression of CCK-AR and CCK-BR protein in mouse DRGs. GAPDH is shown as loading control. Representative western blots are shown from at least three independent experiments. **b**, co-localization of CCK-BR (*Red*) with three markers (*Green*) (CGRP, IB4, and NF-200) in naïve mouse DRGs. Arrows show the co-localization. Scale bars: 30 μm. **c, d**, time course of changes in *I*_A_ amplitude mediated by 100 nM CCK-8 in the presence of either devazepide (1 μM, *n* = 10, **c**) or LY225910 (1 μM, *n* = 8, **d**). *Inset*s show the exemplary current traces. The Arabic numerals indicate the relative points utilized for exemplary current traces. **e,** bar graph showing that treatment of DRG neurons with LY225910, instead of devazepide, abrogated the CCK-8-induced *I*_A_ response. **f,** bar graph showing that application of BC264 (0.1 μM, *n* = 7) or CCK-4 (300 nM, *n* = 7), but not GW5823 (5 μM, *n* = 9), significantly decrease *I*_A_ in small DRG neurons. *p < 0.05 and **p < 0.01 vs. control

The CCK-AR and CCK-BR have been identified as the endogenous receptors for CCK-8 [27]. To determine which one is involved in the CCK-8–induced *I*_A_ reduction, we first examined the protein profile and subcellular expression of these receptors in mouse DRGs. Immunoblot analysis revealed that only CCK-BR (predicted size of 80 kDa), but not CCK-AR (predicted size of 95 kDa), were endogenously expressed (Fig. 2a). Protein lysates prepared from the gallbladder of the same mice were used as a positive control. Small unmyelinated sensory neurons have been classified into isolectin B4 (IB_4_)-positive (non-peptidergic) subset and peptidergic (IB_4_ negative) subset expressing calcitonin gene-related peptide (CGRP), while large neurons in myelinated A-fibers express neurofilament 200 (NF200). We analyzed the CCK-BR expression in DRGs subsets by coimmunostaining of CCK-BR with the mentioned markers. CCK-BRs were found to be heavily colocalized with IB_4_ and CGRP, and less with neurofilament-200 (NF-200), a marker for myelinated A-fibers (Fig. 2b). Next, we determined the participation of CCK-BR in the effect of CCK-8 on *I*_A_. While the CCK-8 mediated reduction of currents was not affected by the presence of 1 μM of the CCK-AR antagonist devazepide (decreased by 29.3 ± 3.7%, *n* = 10, Figs. 2c and e), such effect was completely abolished in the presence of 1 μM of the CCK-BR antagonist LY225910 (decreased by 4.7 ± 0.9%, *n* = 10, Figs. 2d and e). This evidence undoubtably indicates that the CCK-BR, but not the CCK-AR, is involved in the CCK-8-induced *I*_A_ reductions. Furthermore, application of 0.1 μM BC-264, a selective CCK-BR agonist, significantly decreased *I*_A_ by 30.9 ± 2.7% (*n* = 7, Fig. 2f), while the selective CCK-AR agonist GW5823 (5 μM) elicited no such effect (decreased by 3.1 ± 1.2%, *n* = 9, Fig. 2f). Since it is known that both CCK-4 and CCK-8 are active forms of CCKs in the nervous system [28, 29], we also test whether application of another selective CCK-BR agonist CCK-4 affects *I*_A_ in small-sized DRG neurons. Indeed, CCK-4 at 300 nM significantly decreased *I*_A_ by 32.2 ± 4.9% (*n* = 7). These findings further support the conclusion that the CCK agonist driven *I*_A_ decrease was mediated specifically by CCK-BR in small-sized DRG neurons.

### The CCK-BR-mediated I_A_ decrease requires the βγ subunits (G_βγ_) of go-protein

**Fig. 3:**
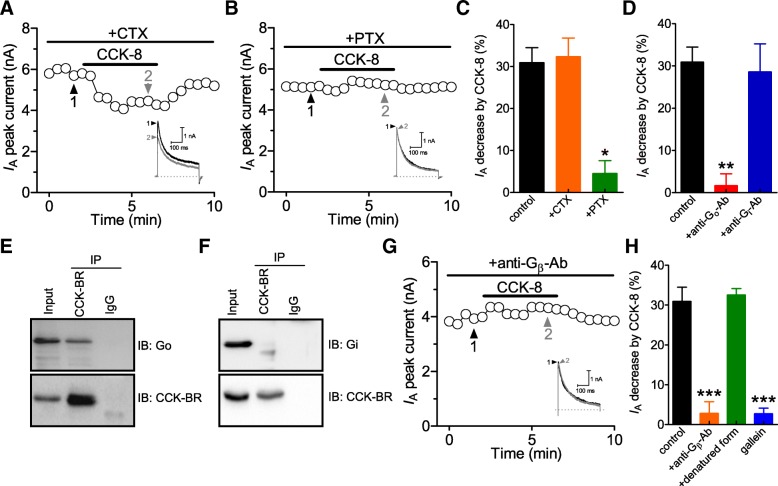
The CCK-BR-mediated *I*_A_ decrease requires the βγ subunits (G_βγ_) of G_o_ protein. **a, b**, time courses showing the effect of 100 nM CCK-8 on the peak amplitude of *I*_A_ in cells pretreated with CTX (500 ng/ml, 16 h pretreatment, *n* = 11, **a**) and PTX (200 ng/ml, 16 h pretreatment, *n* = 7, **b**), respectively. *Inset*s indicate the exemplary current traces. The Arabic numerals indicate the relative points utilized for exemplary current traces. **c,** summary of results showing the effect of CCK-8 (100 nM) on *I*_A_ in CTX- or PTX-pretreated DRG neurons. **d,** bar graph indicating the effect of CCK-8 (100 nM) on *I*_A_ in cells dialyzed with an antibody specific against Gα_i_ (anti-G_i_-Ab, 2 μg/ml, *n* = 8) and anti-G_o_-Ab (2 μg/ml, *n* = 9), respectively. **e, f,** the association of Gα_o_ with the CCK-BR. Co-IP studies reveal that Gα_o_ (**e**), but not Gα_i_ (**f**), interacts with CCK-BR in mouse DRG extracts. Representative western blots are shown from at least three independent experiments. **g,** time course showing the effect of 100 nM CCK-8 on *I*_A_ in the presence of anti-G_β_-Ab (intracellular application, 2 μg/ml). *Inset*s show exemplary current traces. The Arabic numerals indicate the relative points utilized for exemplary current traces. **h,** bar graph showing that intracellular application of gallein (10 μM, *n* = 9) or anti-G_β_-Ab (*n* = 9), but not the denatured form of anti-G_β_-Ab (*n* = 8), abolished the CCK-8-induced *I*_A_ decrease. *p < 0.05, **p < 0.01 and ***p < 0.001 vs. control

CCK-BR coupled to heterotrimeric G-proteins, which are key transducers to control numerous cellular processes [3]. We next examined actions of different subtypes of G-proteins in the CCK-BR-mediated *I*_A_ modulation. Inactivation of G_s_ by pre-treating DRG neurons with cholera toxin (CTX, 500 ng/ml) had no significant effects on the CCK-8-induced *I*_A_ decrease (decreased by 32.3 ± 3.6%, *n* = 11, Fig. 3a and c). Contrastingly, pre-treating cells with pertussis toxin (PTX, 200 ng/ml) to inactivate G_i/o_ abrogated the CCK-8-induced response (decreased by 3.9 ± 2.2%, *n* = 7, Fig. 3b and c). This CCK-BR-induced PTX-sensitive, but CTX-insensitive decrease in *I*_A_, indicated the involvement of G_i/o_, but not G_s_ in the signaling cascade. Further, dialysis of cells with an antibody specifically against Gα_o_ (2 μg/ml) blocked the effect of CCK-8 on *I*_A_ reduction (decreased by 1.5 ± 2.9%, *n* = 8, Fig. 3d), whereas a Gα_i_-specific antibody (2 μg/ml) had no such effect (decreased by 27.6 ± 4.7%, *n* = 9, Fig. 3d). Together, these findings suggest that Gα_o_-protein mediates the response to CCK-8. Moreover, we found that endogenous Gα_o_ (Fig. 3e), but not Gα_i_ (Fig. 3f), was co-immunoprecipitated with an antibody against CCK-BR from DRG tissues, indicating that the CCK-BR and the Gα_o_ subunit form a complex in situ. Further, intracellular application of a G_β_-specific antibody abrogated the CCK-8-induced *I*_A_ reduction (decreased by 3.3 ± 3.5%, *n* = 9; Figs. 3g and h), while its denatured form did not elicit such effects (decreased by 32.5 ± 1.3%, *n* = 8; Fig. 3h). Similar results were obtained with a specific G_βγ_ inhibitor, gallein. Pretreatment of cells with gallein (10 μM) completely abolished the CCK-8-induced *I*_A_ reduction (decreased by 2.7 ± 0.9%, *n* = 9; Fig. 3h). Thus, the G_βγ_ subunit of G_o_-protein is also required for the CCK-BR-mediated *I*_A_ reductions.

### The CCK-BR–mediated *I*_A_ decrease requires PI3K, but independently of Akt

**Fig. 4:**
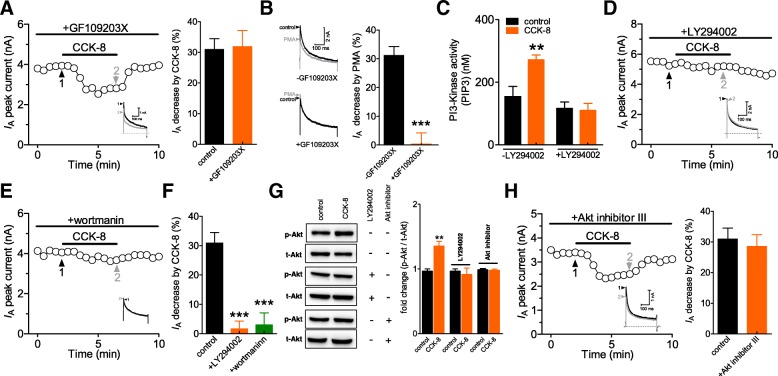
The CCK-BR-mediated *I*_A_ decrease requires PI3K, but not Akt. **a**, time course of changes in *I*_A_ amplitude (*left*) and summary data (*right*) showing the effects of 100 nM CCK-8 on *I*_A_ in the presence of GF109203X (1 μM for 30 min, *n* = 9). *Inset*s show the representative current traces. The numbers on the plot indicate which points were used for sample traces. **b,** representative traces (*left*) and bar graph (*right*) indicating the effect of 5 μM PMA on *I*_A_ in the absence (*n* = 5) or presence (*n* = 9) of 1 μM GF109203X. **c,** pretreating cells with 20 μM LY294002 prevented the CCK-8-induced increase in PI3K activity. The experiments were conducted in triplicate and yielded with similar results. **d, e,** time course showing the effect of CCK-8 (100 nM) on *I*_A_ in the presence of LY294002 (20 μM for 30 min, **d**) or wortmannin (1 μM for 30 min, **e**). *Inset*s show exemplary current traces. The Arabic numerals indicate the relative points utilized for exemplary current traces. **f**, bar graph showing the effects of CCK-8 on *I*_A_ in the presence of LY294002 (*n* = 8) or wortmannin (*n* = 10) indicated in panels **d** and **e** respectively. **g**, CCK-8 induced a significant increase in the phosphorylated Akt (*p*-Akt) in DRG cells. This effect was abrogated by pretreating cells with LY294002 (20 μM for 30 min) or the Akt inhibitor III (10 μM for 30 min). **h,** *left:* representative traces and time course indicating the inhibitory effects of 100 nM CCK-8 on *I*_A_ in the presence of Akt inhibitor III (10 μM). *Inset*s show exemplary current traces. The Arabic numerals indicate the relative points utilized for exemplary current traces. *Right:* bar graph showing that treatment of DRG neurons with Akt inhibitor III (*n* = 9) had no effect on the CCK-8–induced *I*_A_ response. **p < 0.01 and ***p < 0.001 vs. control

In view of the fact that protein kinase C (PKC) has been shown to act downstream of G_βγ_ [30] and regulate *I*_A_ superficial dorsal horn neurons [31], we pre-incubated cells with PKC inhibitors and found that pretreating with GF109203X (1 μM) did not affect the CCK-8-induced *I*_A_ response (decreased by 31.8 ± 4.3%, *n* = 9, Fig. 4a) while pre-incubation of cells with GF109203X substantially blocked the PKC activator PMA (phorbol 12-myristate 13-acetate)-induced *I*_A_ reduction (5 μM, *n* = 9, Fig. 4b). Previous studies have highlighted the critical role of PI3K/Akt cascades in G_βγ_–mediated responses [32]. Thus, we investigated whether the inhibitory effect of CCK-8 on *I*_A_ was PI3K/Akt-dependent. We found that CCK-8 application significantly induced PI3K activation and that pretreating cells with the PI3K inhibitor LY294002 at 20 μM abolished this effect (Fig. 4c). Consistently, pre-treatment with the PI3K inhibitor LY294002 (20 μM) (decreased by 1.9 ± 2.7%, *n* = 8, Fig. 4d and f) or wortmannin (1 μM) (decreased by 2.7 ± 3.5%, *n* = 10, Figs. 4e and f) also prevented the CCK-8 effects on *I*_A_, indicating the involvement of PI3K in the CCK-BR–mediated *I*_A_ decrease. Further, we examined whether the CCK-8 action is also mediated by Akt, a major downstream target of the PI3Ks [33]. We measured the Akt activity in DRG cells and found that 100 nM CCK-8 significantly increased the phosphorylated Akt (*p*-Akt) level, while the total Akt (*t*-Akt) remained unchanged (Fig. 4g). This effect was abrogated by the Akt inhibitor III (10 μM, Fig. 4g). To further determine the involvement of Akt in the modulation of *I*_A_ by CCK-8, cells were pretreated with Akt inhibitor III prior to CCK-8 application. Interestingly, in the presence of 10 μM Akt inhibitor III, CCK-8 at 100 nM still induced a significant decrease in *I*_A_ (decreased by 28.5 ± 3.8%, *n* = 9, Fig. 4h), revealing that the CCK-8–induced *I*_A_ decrease was mediated by PI3K, but independently of Akt.

### CCK-8 attenuates *I*_A_ through c-Src-dependent JNK pathway

**Fig. 5:**
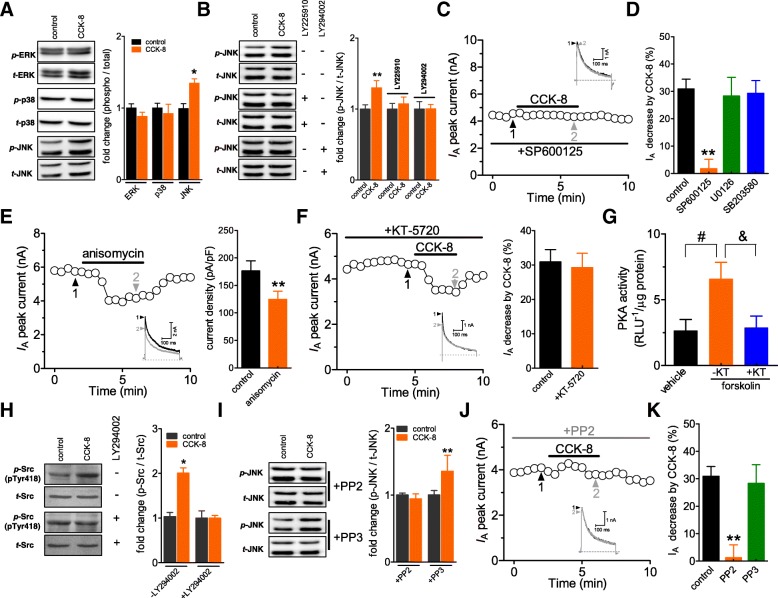
CCK-8 attenuates *I*_A_ through Src-dependent JNK pathway. **a** CCK-8 increased the protein levels of phosphorylated JNK (*p*-JNK), with no significant changes in *p*-p38 and *p*-ERK. Representative western blots are shown from at least three independent experiments. **b** activation of JNK by CCK-8 is via CCK-BR and requires PI3K activity. Pretreatment of cells with LY225910 (1 μM) or LY294002 (20 μM) abolished CCK-8–induced JNK phosphorylation. Representative western blots are shown from at least three independent experiments. **c**, time course showing the effect of 100 nM CCK-8 on *I*_A_ in the presence of SP600125. *Inset*s show representative current traces. The Arabic numerals indicate the relative points utilized for exemplary current traces. **d** bar graph showing the effects of CCK-8 (100 nM) on *I*_A_ in the presence of SP600125 (10 μM, *n* = 7), U0126 (1 μM, *n* = 9), and SB203580 (10 μM, *n* = 11), respectively. **e** Time course (*left panel*) and summary of results (*right panel*) indicating that application of anisomycin (25 ng/ml, *n* = 11) significantly decreased peak *I*_A_ amplitude in small DRG neurons. **f** time course (*left panel*) and summary of results (*right panel*) indicating the effects of 100 nM CCK-8 on *I*_A_ in the presence of KT-5720 (1 μM for 30 min, *n* = 10). *Inset*s show exemplary current traces. The Arabic numerals indicate the relative points utilized for exemplary current traces. **g** bar graph showing the effects of 20 μM forskolin on PKA activity in DRG cells pretreated with KT-5720 (1 μM). The experiments were conducted in triplicate and yielded with similar results. **h** the level of phosphorylated Src (pTyr418, *p*-Src) increased following treatment with CCK-8 (100 nM). This effect was abolished by the PI3K inhibitor LY294002 (20 μM for 30 min). Representative western blots are shown from at least three independent experiments. **i** CCK-8-induced JNK activation is blocked by the Src inhibitor PP2 (10 μM). PP2 or its inactive structure analog PP3 (10 μM) was pre-administered for 30 min before CCK-8 addition. Representative western blots are shown from at least three independent experiments. **j** time course indicating the effects of CCK-8 on *I*_A_ in the presence of 10 μM PP2. *Inset*s show exemplary current traces. The Arabic numerals indicate the relative points utilized for exemplary current traces. **k** bar graph indicating that application of PP2 (10 μM for 30 min, *n* = 12), but not PP3 (10 μM for 30 min, *n* = 8), abolished the CCK-8-induced *I*_A_ decrease. * *p* < 0.05 and ** *p* < 0.01 vs. control; ^#^ *p* < 0.05 vs. vehicle; ^&^ *p* < 0.05 vs. forskolin without KT-5720

Mitogen-activated protein kinases (MAPKs), composing a family of protein kinases that play pivotal roles in mediating pain sensitivity [34], were shown to regulate neuronal *I*_A_ [31]. Thus, it was of interest to the current study to examine whether the MAPK cascades would be involved in the CCK-BR-induced response. Immunoblot analysis indicated that the exposure of DRG cells to CCK-8 (100 nM) significantly increased the expression of phosphorylated c-Jun N-terminal kinase (*p*-JNK*)*, while the protein levels of total JNK (*t*-JNK), *p*-p38 as well as *p*-ERK activity remained unchanged (Fig. 5a). Blockade of CCK-BR with LY225910 (1 μM), as well as pretreating cells with the PI3K antagonist LY294002 (20 μM), eliminated the CCK-8–induced JNK activation (Fig. 5b). These findings reveal that the PI3K-mediated JNK signaling was involved in the CCK-8–induced effects. Next, we pretreated cells with 10 μM SP600125, a specific JNK inhibitor, and found that SP600125 abrogated the *I*_A_ decrease induced by CCK-8 (decreased by 0.7 ± 1.9%, *n* = 7, Figs. 5c and d). Contrastingly, U0126 (1 μM), a MAPK/ERK (MEK) inhibitor, as well as the p-38 inhibitor SB203580 (10 μM), elicited no such effects (U0126: decreased by 28.3 ± 5.5%, *n* = 9; SB203580: decreased by 29.3 ± 3.8%, *n* = 11; Fig. 5d). As complementary support, the application of the JNK agonist anisomycin (25 ng/ml) to DRG neurons induced a significant decrease in *I*_A_ amplitude (decreased by 32.7 ± 3.9%, *n* = 11, Fig. 5e).

Protein kinase A (PKA) was involved in the regulation of Kv channel currents [24, 31] and has been suggested to mediate the crosstalk between the PI3K and MAPK pathways [35]. Thus, we asked whether the JNK-dependent regulation of *I*_A_ by CCK-8 required PKA. Pretreatment of cells with the PKA inhibitor, KT-5720 (1 μM), had no significant effect on the CCK-8–induced *I*_A_ decrease (decreased by 29.1 ± 3.2%, *n* = 10, Fig. 5f), while the administration of KT-5720 (1 μM) blocked 20 μM forskolin-induced PKA activation (Fig. 5g), indicating a PKA-independent mechanism involved in CCK-BR-mediated response. Src kinase (Src) has been demonstrated to activate JNK pathways [36, 37]. We assayed the cellular Src activity in DRG cells treated with CCK-8. Figure 5h illustrates that phosphorylated Src (pTyr418, *p*-Src) increased following treatment with CCK-8 (100 nM), whereas the protein levels of total Src (*t*-Src) was unchanged (Fig. 5h). This effect was abolished by the PI3K inhibitor LY294002 (20 μM, Fig. 5h). Moreover, cells were treated with the Src-specific inhibitor PP2 (10 μM) prior to CCK-8 exposure, and the JNK activation was monitored. Pretreatment of the cells with the Src inhibitor PP2 (10 μM) completely abolished CCK-8-induced JNK activation, while the inactively structural analog PP3 (10 μM) elicited no such effect (Fig. 5i). Consistent with this, pretreatment of cells with PP2 (10 μM) (decreased by 0.6 ± 2.7%, *n* = 12, Fig. 5j and k), but not PP3 (decreased by 26.9 ± 3.1%, *n* = 8, Fig. 5k), completely abolished the CCK-8-induced *I*_A_ decrease. Collectively, these results suggest that Src, but not PKA, mediated the signaling between PI3K and JNK in the CCK-BR-mediated *I*_A_ response.

### Activation of CCK-BRs induces DRG neuronal hyperexcitability

**Fig. 6:**
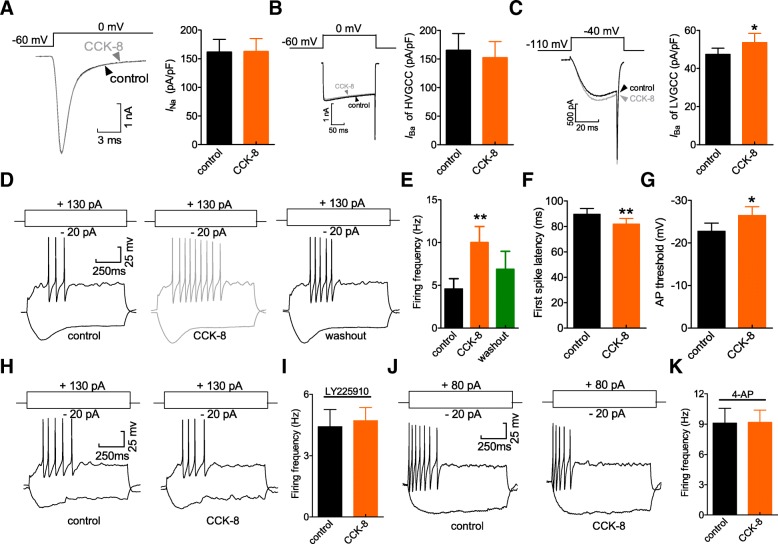
CCK-8 induces neuronal hyperexcitability. **a-c** exemplary current traces (*left*) and bar graph (*right*) indicating the effect of 100 nM CCK-8 on Nav currents (*I*_Na_, *n* = 11, *A*), high-voltage-activated Cav currents (*I*_Ba_ of HVGCC, *n* = 8, **b**), or low-voltage-activated Cav currents (*I*_Ba_ of LVGCC, *n* = 9, **c**), respectively. Either Nav currents or *I*_Ba_ of HVGCC were elicited by a test pulse to 0 mV from a holding potential of − 60 mV. A stepped voltage protocol from − 110 to − 40 mV with a holding potential of − 110 mV was applied to elicit *I*_Ba_ of LVGCC. **d, e** exemplary traces (**d**) and summary of results (**e**, *n* = 17) indicating the effect of CCK-8 (100 nM) on action potential firing rate. Representative traces were recorded when small-sized DRG neurons were subjected to 130 pA current injections. **f, g** CCK-8 at 100 nM significantly shortened first spike latency (**f**) and decreased the AP threshold (**g**) in small-sized DRG neurons (*n* = 17). **h, i** representative traces (**h**) and summary of results (**i**) showing that pretreatment of cells with LY225910 (1 μM, *n* = 12) abolished the 100 nM CCK-8-induced the increase in firing rate. Representative traces were recorded when small-sized DRG neurons were subjected to 130 pA current injections. **j, k** exemplary current traces (**j**) and summary of results (**k**) indicating that application of 4-AP at 5 mM abrogated the 100 nM CCK-8-induced neuronal hyperexcitability (*n* = 12). Representative traces were recorded when small-sized DRG neurons were subjected to 80 pA current injections. *p < 0.05 and **p < 0.01 vs. control

Kv exerts pivotal effects in modulating neuronal excitability in peripheral sensory neurons [38]. To determine the functional roles of the CCK-BR-mediated *I*_A_ response, we determined whether the membrane excitability of DRG neurons would be affected by CCK-8. Bath application of CCK-8 (100 nM) had no significant effects on the whole-cell currents of Nav (*n* = 11, Fig. 6a) and the high voltage-activated (HVA) calcium channel currents (*n* = 8, Fig. 6b) in small DRG neurons, whereas CCK-8 increased LVA (T-type) channel currents by 8.3% (*n* = 9, Fig. 6c). Using an external solution including Z941 (10 μM) to block T-type channels, we found that 100 nM CCK-8 significantly increased action potential (AP) firing in response to 1-s current injection (by 56.6 ± 2.9% compared to control, *n* = 17, Fig. 6d and e). After washout, the firing rate was partially restored (Fig. 6e). Additionally, CCK-8 (100 nM) significantly shortened the first spike latency (Fig. 6f) and decreased AP threshold (*n* = 17, Fig. 6g). The other membrane properties of neuronal excitability, including resting membrane potential, were not significantly changed by 100 nM CCK-8 (not shown). Pretreating neurons with LY225910 (1 μM) abrogated the CCK-8-induced increase in AP firing rate, indicating the CCK-BR involvement (*n* = 12, Fig. 6h). To further verify the CCK-BR-induced neuronal hyperexcitability through *I*_A_ decrease, 4-AP was applied prior to CCK-8. Pre-treatment of DRG neurons with 5 mM 4-AP abrogated the neuronal hyperexcitability induced by 100 nM CCK-8 (*n* = 12, Fig. 6j and k), indicating that the CCK-BR-mediated *I*_A_ decrease subsequently induced neuronal hyperexcitability in small DRG neurons.

### Involvement of A-type channels encoding I_A_ in CCK-induced pain hypersensitivity

**Fig. 7:**
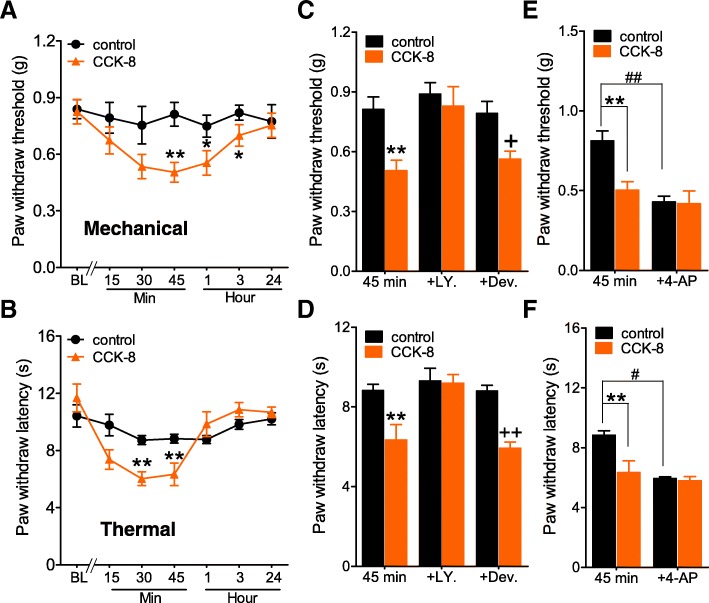
Involvement of peripheral CCK-BR activation in pain hypersensitivity. **a,b** intraplantar injection (i.p.l.) of CCK-8 (50 ng) induced mechanical (**a**) and heat (**b**) pain hypersensitivity. *p < 0.05, **p < 0.01, CCK-8 injection vs. vehicle. **c, d** pretreatment of LY225910 (0.5 μg, i.p.l., **c**), but not devezepide (1 μg, i.p.l., **d**), significantly attenuated CCK-8-induced mechanical hypersensitivity and thermal hyperalgesia. **e, f** intraplantar pre-injection of 4-AP (25 nmol) occluded the thermal and mechanical hypersensitivity induced by CCK-8 (50 ng). *p < 0.05, **p < 0.01, CCK-8 injection vs. vehicle; ^+^ *p* < 0.05 and ^++^ *p* < 0.01, CCK-8 injection vs. CCK-8 + devazepide (Dev.) at 45 min; ^#^*p* < 0.05 and ^##^*p* < 0.01, 4-AP injection vs. vehicle at 45 min. *N* = at least 8 mice for all animal behavior experiments

Further, we determined whether CCK-8 would affect in vivo pain sensitivity in animals. Intraplantar injection of CCK-8 (50 ng) markedly increased pain sensitivity to both mechanical and heating stimuli (Figs. 7a and b). The CCK-8-induced pain hypersensitivity to mechanical or heating stimulation was completely abrogated by intraplantar pretreatment of the CCK-BR antagonist LY225910 (0.5 μg, Figs. 7c and d), but not by the CCK-AR antagonist devazepide (1 μg, Figs. 7c and d). Moreover, intraplantar pretreatment with 4-AP (25 nmol) induced a significant increase in mechanical and heat sensitivity as compared with animals received a saline injection (Figs. 7e and f). Sensitivity assessed after intraplantar injection of CCK-8 showed that CCK-8 did not induce any additive effects to that of 4-AP on mechanical (Fig. 7e) and thermal (Fig. 7f) pain sensitivity, strongly suggesting that CCK-8 and 4-AP likely target molecules in the same cellular signaling pathway in vivo. Collectively, these findings reveal that A-type channels encoding *I*_A_ contribute to the CCK-BR–mediated acute pain hypersensitivity.

## Discussion

**Fig. 8:**
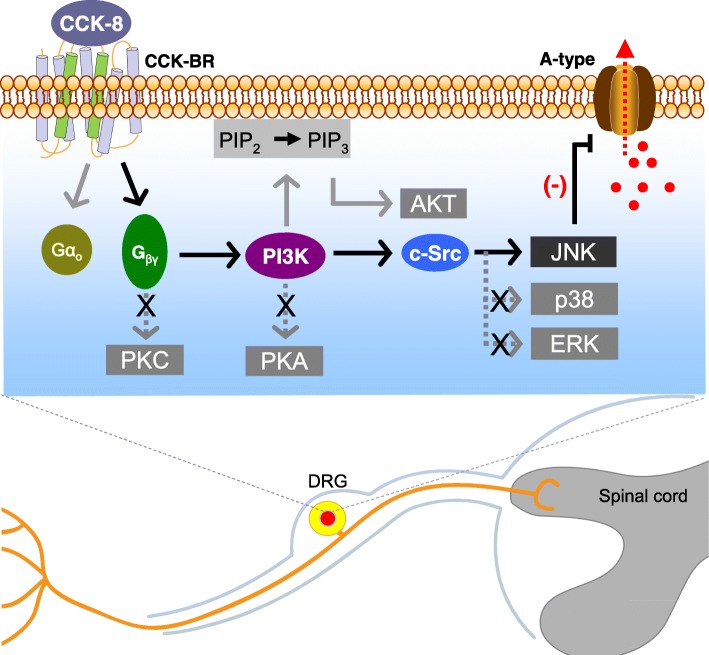
Schematic shows the regulation of CCK-BR on *I*_A_ and the involvement of CCK-8/CCK-BR in pain sensitivity. CCK-8 stimulates the G_o_-protein coupled CCK-BR and thereafter releases the βγ subunits (G_βγ_). The released G_βγ_ subsequently activates PI3K, which decreases the *I*_A_ and induces neuronal hyperexcitability and pain hypersensitivity. PI3K catalyzes the conversion of PtdIns(4,5)P2 (PIP2) to PtdIns(3,4,5)P3 (PIP3), which serves as a second messenger that helps to activate Akt. However, neither PKA/PKC/Akt nor the direct binding of G_βγ_ with A-type channels contributes to the CCK-BR-mediated *I*_A_ response. In mouse DRG neurons, PI3K signaling may activate Src, which then phosphorylates JNK to modulate *I*_A_. Whether the activated *p*-JNK would phosphorylates Kv channels encoding *I*_A_ or in turn stimulated intermediate molecules still needs further examined

The present study provides mechanistic data describing a novel functional role of CCK-8 in modulating transient *I*_A_ in small-sized DRG neurons, without any concurrent effect on *I*_DR_. Based on our findings, we propose a signaling cascade model in which CCK-8–stimulated PI3K recruits the Src-dependent JNK to suppress *I*_A_. This attenuation of *I*_A_ induced by CCK-8 application is mediated by the stimulation of CCK-BR and leads to sensory neuronal hyperexcitability and pain hypersensitivity in mice (see Fig. 8).

The PKC family of isozymes mediates *I*_A_ responses in a cell-type and tissue-specific manner. For instance, activation of group I metabotropic glutamate receptors led to an inhibition of *I*_A_ through a PKC-dependent mechanism in striatal cholinergic interneurons, while in large aspiny neurons activation of PKCα increases *I*_A_. Interestingly, in murine proximal colonic myocytes, the PKC-independent regulation of *I*_A_ has also been reported [39]. In this study, the CCK-8-induced *I*_A_ decrease was independent of PKC and was mediated by PI3K, through the JNK-dependent signaling. These results are supported by previous studies that Kv currents including *I*_A_ recorded from trigeminal ganglion neurons and pancreatic β cells decreased in response to PI3K pathway activation [21, 40]. Interestingly, the activation of PI3K has also been reported to increase *I*_A_ in cultured rat cerebellar granule cells [41]. In addition, PI3K-induced activation of Kv4.3 channels through glucocorticoid-inducible kinase-1 (SGK1) was also reported [42]. Although these discrepancies require further clarification, the regulatory effects of PI3K would be variable in tissues/cell types expressing different A-type channel subunits. Another appropriate alternative hypothesis is that the stimulatory PI3K can also phosphorylate an intermediate protein that in turn down-regulates *I*_A_ in small-sized DRG neurons. Furthermore, different splice variants of KChIP auxiliary subunits of *I*_A_ channels can engender different, even opposing, modulation of Kv4 channel currents [43].

A known target of G_βγ_ is PI3K [33]. In contrast to many other common G_βγ_-dependent PI3K signal transduction events, the CCK-8–induced PI3K dependent *I*_A_ attenuation isnot mediated by Akt, as demonstrated by the specific inhibition of Akt with pharmacological agents. Interestingly, previous studies have shown that Akt both negatively and positively regulates Kv4 [44, 45], which forms one of the major components mediating *I*_A_. For example, Akt down-regulates the activity of Kv4 channels in cultured cerebellar granule cells of rats [45]; in the same neurons, a different study demonstrates that enhanced Akt activity is required for *I*_A_ amplification and Kv4.2 induction [44]. This Akt-dependent stimulation of Kv4 also occurs in the arcuate nucleus [46]. Thus, it appears that Akt differentially regulates the activity of Kv4 channels in a tissue-specific manner. In our study, the CCK-BR-mediated *I*_A_ response was found to be independent of Akt; therefore, we went on to investigate what mediating PI3K signals to suppress *I*_A_ in DRG neurons. Considerable in vivo and in vitro studies indicate that ERK plays pivotal roles in neuropathic pain [34, 47]. Phosphorylated ERK is elevated in DRG cells following peripheral nerve injury [48]. Intrathecal application of ERK inhibitors reduces the pain behavior associated with nerve injury [49]. Moreover, one of the most convincing evidence comes from the direct phosphorylation of the pore-forming channel subunit of Kv4.2 by ERK [50] that determine a downregulation of *I*_A_ in superficial dorsal horn neurons [31]. Contrastingly, antagonism of ERK completely abrogated *I*_A_ increase induced by dopamine in lateral pyloric neurons [51]. However, we found that the CCK-8-induced decrease of *I*_A_ was unlikely induced by ERK phosphorylation, because the CCK-8 application did not change the ERK activity in DRG cells, whereas the levels of *p*-JNK were significantly increased. Moreover, the MAPK/ERK inhibitor did not affect the CCK-8–induced *I*_A_ response. Our findings suggested that PI3K stimulated JNK in DRG neurons and that this signaling is essential for the CCK-BR–mediated *I*_A_ response. Our results showed that 1) application of the JNK inhibitor SP600125, but not the p38 MAPK inhibitor SB203580 or the MAPK/ERK (MEK) inhibitor U016, blocked the CCK-8–induced *I*_A_ decrease and 2) antagonism of PI3K blocked CCK-BR–mediated JNK activation. Consistent with these findings, the increased activity of JNK in ventricular myocytes markedly decreased the amplitude of transient outward K^+^ current density [52]. These observations are in line with an earlier study showing a C-reactive protein (CRP)-induced modulation of intracellular JNK and interactions with voltage-activated K^+^ channels [53].

Up till now, it is still relatively unclear how PI3K activates JNK. It has been established that that PI3K may stimulate PKA, subsequently activating the downstream MAPK pathway [35]. In the present study, activation of CCK-BR did not influence the PKA activity in DRG cells, indicating some other mechanisms, but not of PKA, mediate the crosstalk between PI3K and JNK signaling. Src kinases are downstream of PI3K and can facilitate JNK activity [37], suggesting possible crosstalk between PI3K and JNK signaling. In support of this observation, the current study demonstrated that the Src kinase inhibitor PP2 blocked the CCK-8–induced JNK activation. The blockade of PI3K also abolished the CCK-BR-mediated increase in Src activity, indicating that PI3K may modulate the JNK pathway through c-Src. Therefore, it is likely that CCK-8–activated PI3K recruits Src to up-regulate JNK activity, and thereby regulating CCK-BR–mediated *I*_A_ response in DRG neurons.

*I*_A_, encoded by A-type K^+^ channels, is important determinants of both the delay of spike onset (first spike latency) and the decrease in the firing frequency [17]. Acute decreases in *I*_A_ in sensory neurons cause robust increases in neuronal excitability, [38] and may increase the responsiveness to nociceptive stimulation and contribute to mechanical hypersensitivity and thermal hyperalgesia [54]. Genetic studies have firmly established a prominent role for A-type channels in amplifying nociceptive signals in the periphery and in contributing to central sensitization in the spinal dorsal horn [17, 19, 55]. Further, recent evidence has suggested that modulation of peripheral A-type channels influences somatic and visceral nociceptive inputs and thus an increase of A-type channel currents results in significant anti-nociception in a variety of animal neuropathic pain models [19]. In the current study, consistently with the CCK-8-induced *I*_A_ decrease, activation of CCK-BR led to increased excitability in DRG neurons with increased spike frequency and shortened first-spike latency, both of which are major parameters determining the timing of neurotransmitter release, and hence pain transmission [56]. In addition, acute mechanical hypersensitivity and thermal hyperalgesia mediated by CCK-BR can be occluded by the A-type K^+^ channel blockade. As such, our findings are supportive of the reasonable assumption that nociceptive actions of CCK-BR are mediated, at least in part, through the JNK-dependent reduction of *I*_A_. Our present results are, indeed, in accordance with previous studies that CCK-8 might induce pro-nociceptive actions [8, 9]. Intrathecal inhibition of JNK, a key modulator *I*_A_ in the present study, has been found to attenuate the CCI-induced mechanical allodynia and thermal hyperalgesia in rats [57]. Following spinal nerve ligation (SNL), phosphorylated JNK in small-sized DRG neurons have been found to be greatly increased [58] and the intrathecal infusion of JNK inhibitor can reverse mechanical but not thermal hypersensitivity [59].

## Conclusions

Collectively, this study found that CCK-8 decreases *I*_A_ through the G_βγ_-mediated PI3K/Src/JNK pathway. This mechanism occurred via CCK-BR and mediated the neuronal hyperexcitability in peripheral sensory neurons and pain hypersensitivity in mice. Modulation of *I*_A_ by CCK-8 in peripheral sensory neurons is of particular interest. The identification of CCK-BR-mediated molecular mechanisms contributing to pain hypersensitization may offer insights into opportunities for analgesic pharmacotherapy.

## Data Availability

All data and materials generated in this study are available upon request.

## Abbreviations

4-AP: 4-aminopyridine
CCK-8: Cholecystokinin-8
CCK-BR: CCK type B receptor
CTX: Cholera toxin
ERK: Extracellular regulated protein kinases
GDP-β-S: Guanosine-5′-O-(2-thiodiphosphate)
GPCR: G-protein coupled receptor
G_βγ_: G-protein βγ subunit
*I*_A_: A-type K^+^ current
*I*_DR_: Sustained delayed rectifier K^+^ current
JNK: c-Jun N-terminal kinase
MAPK: Mitogen-activated protein kinase
PI3K: Phosphatidylinositol 3-kinase
PKA: Protein kinase A;
PKC: Protein kinase C
PTX: Pertussis toxin
